# Gene-body hypermethylation controlled cryptic promoter and miR26A1-dependent EZH2 regulation of TET1 gene activity in chronic lymphocytic leukemia

**DOI:** 10.18632/oncotarget.20668

**Published:** 2017-09-06

**Authors:** Pradeep Kumar Kopparapu, Mohammad Hamdy Abdelrazak Morsy, Chandrasekhar Kanduri, Meena Kanduri

**Affiliations:** ^1^ Department of Clinical Chemistry and Transfusion Medicine, Institute of Biomedicine, Sahlgrenska Academy, Gothenburg University, Gothenburg, Sweden; ^2^ Department of Medical Biochemistry and Cell Biology, Institute of Biomedicine, Sahlgrenska Academy, Gothenburg University, Gothenburg, Sweden

**Keywords:** gene-body hypermethylation, chronic lymphocytic leukemia, cryptic promoters, EZH2 and *TET1* gene

## Abstract

The Ten-eleven-translocation 1 (TET1) protein is a member of dioxygenase protein family that catalyzes the oxidation of 5-methylcytosine to 5-hydroxymethylcytosine. TET1 is differentially expressed in many cancers, including leukemia. However, very little is known about mechanism behind TET1 deregulation. Previously, by characterizing global methylation patterns in CLL patients using MBD-seq, we found *TET1* as one of the differentially methylated regions with gene-body hypermethylation. Herein, we characterize mechanisms that control *TET1* gene activity at the transcriptional level. We show that treatment of CLL cell lines with 5-aza 2´-deoxycytidine (DAC) results in the activation of miR26A1, which causes decrease in both mRNA and protein levels of EZH2, which in turn results in the decreased occupancy of EZH2 over the *TET1* promoter and consequently the loss of TET1 expression. In addition, DAC treatment also leads to the activation of antisense transcription overlapping the *TET1* gene from a cryptic promoter, located in the hypermethylated intronic region. Increased expression of intronic transcripts correlates with decreased *TET1* promoter activity through the loss of RNA Pol II occupancy. Thus, our data demonstrate that TET1 gene activation in CLL depends on miR26A1 regulated EZH2 binding at the *TET1* promoter and silencing of novel cryptic promoter by gene-body hypermethylation.

## INTRODUCTION

Aberrant DNA methylation profiles are well documented epigenetic alterations that are implicated in several disorders including hematopoietic malignancies. The DNA methylation pattern across the genome is a culmination of a balance between DNA methylation and demethylation, which is brought by dynamically regulated functional interplay between DNA methyl transferases (DNMTs) and Ten-Eleven Translocation protein family (TETs) [1]. Active demethylation of 5-methyl cytosines is implemented by the TET family proteins (TET 1, TET2 and TET3) into 5-hydroxymethyl cytosines, which are subject for further oxidation into 5-formylcytosine and 5-carboxycytosine in a sequential manner [2]. Passive demethylation takes place as a consequence of impaired replication-dependent methylation by DNMT1 where TET proteins would potentiate such passive demethylation [3, 4]. Loss-of-function mutations encountering the TET proteins in lymphoid and myeloid malignancies have been reported especially *TET2*, while less is known about *TET1* and *3* [5, 6]. *TET1* was identified as a fusion partner of mixed lineage leukemia (MLL) gene in AML patients [7]. TET1 has been shown to be overexpressed and downregulated in different cancers and lymphoma patients compared to normal healthy controls [8–11]. TET1 is not only regulated at the genetic level, but also regulated epigenetically as it has been shown to have impaired expression in non-Hodgkin B cell lymphoma (B-NHL) due to promoter hypermethylation [12]. Since cancer genome sequencing analyses rarely identified mutations in the *TET1* coding sequences in hematologic cancers [13], its exact function in normal and malignant hematopoiesis remained unexplored until recent studies reported key roles of TET1 in hematopoietic transformation [14].

Chronic lymphocytic leukemia (CLL) is a common incident in the west and characterized by diverse clinical behavior and heterogeneous disease course [15]. Recent advancements in genetic and epigenetic studies, aided by next-generation sequencing methods, resulted in identifying several markers allowing better stratification of CLL patients for prognosis. However, despite of tremendously improved therapeutic options, CLL still remains as an incurable disease with patients having very shorter survival. Previously, using high-resolution 27K/450K methylation arrays in CLL, we analyzed the global methylation profiles between well-characterized prognostic groups such as IGHV mutated and unmutated CLL subsets [16–18] and identified large number of differentially methylated genes with prognostic implications for the CLL prognostic subgroups.

Recently, our comprehensive methylation analysis by Methyl-CpG-binding domain protein enriched genome-wide sequencing (MBD-Seq), identified several differentially methylated regions, including promoter elements and gene-body regions [19]. It is very well known that promoter methylation correlates with gene silencing, whereas gene-body hypermethylation has recently been shown to correlate with gene activation [20, 21]. Based on recent evidence [22] it is proposed that gene-body hypermethylation may help in maintaining the activity of corresponding promoter by repressing aberrant transcription from cryptic promoters within the gene-body. In this investigation, we were interested in understanding the functional role of gene-body hypermethylation in gene activation using TET1 as model system. Herein we demonstrate that TET1 gene-body hypermethylation represses a cryptic promoter activity located within the intronic region and treatment with demethylation agent DAC results in activation of cryptic promoter and subsequent inactivation of TET1. Importantly, along with gene-body hypermethylation we also show that TET1 expression is dependent on EZH2 occupancy over promoter region.

## RESULTS

### Promoter hypomethylation, gene-body hypermethylation correlates with *TET1* expression in CLL

**Figure 1:**
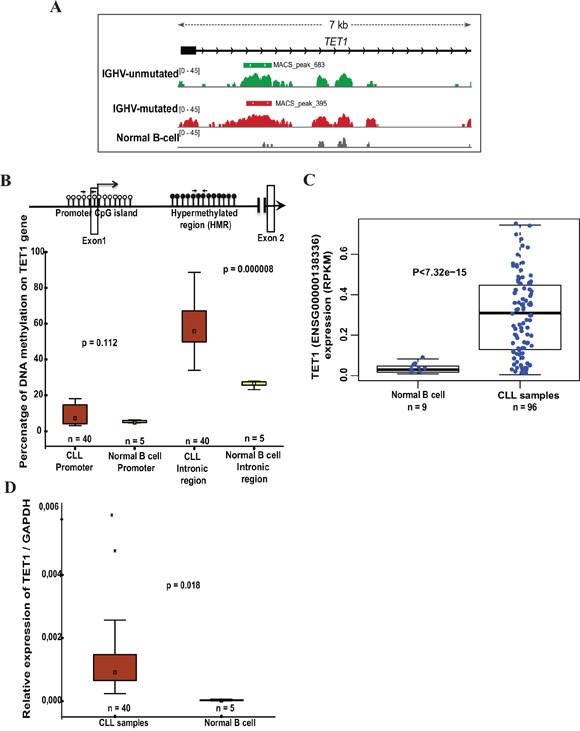
DNA methylation status and *TET1* expression levels in CLL samples and normal B cell controls **(A)** The illustration represents the methylated peaks enriched regions from IGHV-unmutated and IGHV-mutated CLL samples compared to normal B cell control sample with a p value <0.00001 located between exon1 and exon2 of *TET1* gene based on MDB sequencing data. **(B)** Schematic model illustrating the unmethylated CpG sites at promoter region (represented by white-filled lollipops), black-filled lollipops representing methylated CpG sites on gene body intronic region and location of pyrosequencing primer sets used for analyzing DNA methylation status. Below is the box plot showing the percentage of DNA methylation on *TET1* promoter and gene body hypermethylated region between CLL samples and normal B cell samples assessed by pyrosequencing. **(C)** Box plot showing the differential expression of *TET1* analyzed based on independent published RNA sequencing data on 96 CLL patient samples [23]. **(D)** Box plots showing the expression of TET1 in CLL patient samples (n= 40) compared to the normal B cell samples (n=5) using RT-qPCR analysis.

In our recent MBD-seq analysis of CLL patients [19], we observed that *TET1* gene-body (Intronic region) but not its promoter region showed hypermethylation in CLL patient samples compared to normal healthy sorted B cell samples. Both IGHV mutated and unmutated CLL prognostic groups showed significantly hypermethylated specific peak in the *TET1* intronic region compared to normal sorted B cell sample (Figure 1A). When the global methylation data was correlated with the published RNA-seq data in CLL samples [23], we found *TET1* among the top genes showing gene-body hypermethylation and significant expression compared to normal B cells [19]. By performing pyrosequencing on additional 40 CLL samples and 5 normal healthy age-matched sorted B cell samples, we further validated methylation status of the *TET1* gene-body and its promoter regions (20 IGHV mutated + 20 IGHV unmutated samples) (Figure 1B). Consistent with the MBD-seq data, pyrosequencing data also showed hypomethylation of the *TET1* promoter in both CLL and normal B cells, whereas hypermethylation of *TET1* gene-body in all CLL samples (n=40; median 55.7%) compared to normal B cell samples (n=5; median 25%) (Figure 1B). No significant difference was observed in the percentage of DNA methylation between IGHV mutated (n=20; median 55%) and unmutated (n=20; median 56.7%) prognostic groups (Supplementary Figure 1A).

According to our analysis based on the published RNA seq data in CLL [23], *TET1* gene showed significant differential expression in CLL samples (n= 96) compared to normal sorted B cells (n =9) (Figure 1C). We further confirmed this differential expression status of *TET1* using RT-qPCR analysis on further 40 CLL samples along with normal healthy sorted B cell samples, used in our pyrosequencing experiments (Figure 1D). As shown in Supplementary Figure 1B and 1C, IGHV mutated and unmutated CLL samples did not show any significant difference in the TET1 gene expression levels in both RT-qPCR analysis and RNA seq data.

### Interplay of gene-body DNA methylation and EZH2 regulates TET1 expression

**Figure 2:**
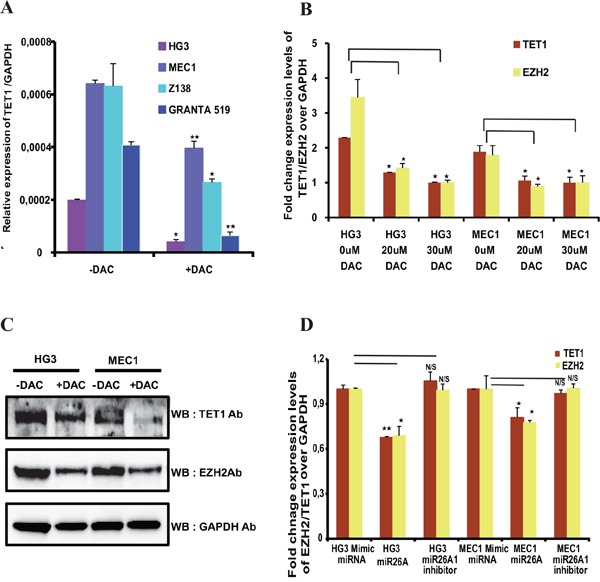
Expression of TET1 and EZH2 after DNA methyl inhibitor treatment and effect of *miR26A1* overexpression **(A)** Relative mRNA expression levels of *TET1* gene over *GAPDH* in both DNA methylation inhibitor (DAC) treated and untreated CLL cell lines (HG3 & MEC1) and MCL cell lines (GRANTA 519 & Z138). **(B)** Relative expression levels of *TET1* and *EZH2* over *GAPDH* in increasing concentrations of DAC treated HG3 and MEC1 cell lines. **(C)** Western blot analysis showing protein levels of TET1 and EZH2 in DAC treated and untreated CLL cell line samples. **(D)** Fold change mRNA expression levels of *TET1* and *EZH2* over *GAPDH* in *miR26A1* and *miR26A1* inhibitor overexpressed CLL cell line samples. The p value significance is indicated as stars compared to control samples (*P < 0.05, **P < 0.005 and NS, not significant).

Since gene-body methylation correlates with *TET1* gene expression, we wanted to explore whether it has any functional link to *TET1* expression. For this purpose, *TET1* expression levels were analyzed in four leukemic cell lines: two CLL cell lines (HG3 and MEC1) and two MCL cell lines (GRANTA 519 and Z138) and all four cell lines express *TET1* gene (Figure 2A). We next treated leukemic cell lines with DNA methyl inhibitor drug (Deoxy-5’-Aza-2 Cytidine known as DAC) for three days and analyzed *TET1* expression levels using RT-qPCR. As shown in the Figure 2A, a significant decrease in *TET1* expression levels in all these cell lines was observed compared to untreated cell line samples. DNA methylation status of *TET1* gene-body was analyzed using pyrosequencing and found that DAC treated samples showed decrease in DNA methylation compared to untreated samples (Supplementary Figure 1D). Thus these observations suggest that gene-body methylation is important and one of the mechanisms required for maintaining TET1 promoter activity.

Previously, we have demonstrated that *miR26A1* microRNA, which targets EZH2 mRNA, is hypermethylated and silenced, leading to higher EZH2 levels in CLL patients [24]. Following DAC treatment, we observed an increase in *miR26A1* expression and a corresponding decrease in EZH2 protein levels in all the four cell lines used in the current study. Since, *miR26A1* was also shown to target *TET1* gene at the post transcriptional level [25], both *EZH2* and *TET1* levels were analysed in the same DAC treated and untreated samples. Dose dependent DAC treatment of HG3 and MEC1 cell lines, resulted in decrease in the mRNA expression levels of both *TET1* and *EZH2* (Figure 2B). Moreover, we also observed decrease in protein expression levels of TET1 and EZH2 upon DAC treatment (Figure 2C). Similarly, decrease in the *TET1* and *EZH2* mRNA levels were observed upon overexpression of *miR26A1* when compared to control microRNA mimics (Figure 2D). Expectedly, overexpression of *miR26A1* inhibitor attenuated the effect of *miR26A1* overexpression on *TET1* and *EZH2* (Supplementary Figure 2A and 2D).

**Figure 3:**
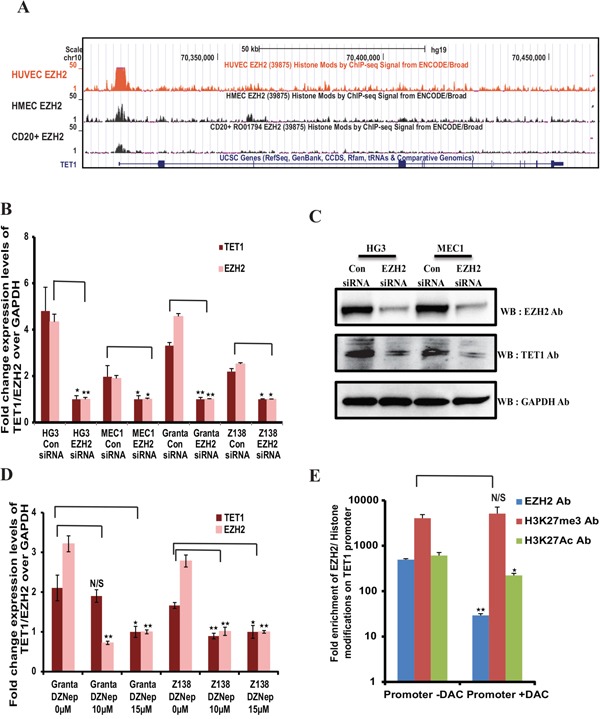
EZH2 dependent expression of *TET1* **(A)** Snapshot of UCSC genome browser in different cell lines from the ENCODE/Broad institute datasets, showing EZH2 binding peaks at *TET1* promoter region. **(B)** Relative expression levels of *TET1* and *EZH2* over *GAPDH* in EZH2 siRNA and control siRNA transfected four leukemic cell lines. **(C)** Western blot analysis showing the protein levels of EZH2 and TET1 in control siRNA and EZH2 siRNA transfected HG3 and MEC1 samples. **(D)** Fold change expression levels of *TET1* and *EZH2* over *GAPDH* in EZH2 downregulated GRANTA 519 and Z138 MCL cell lines, using both siRNA transfections and EZH2 inhibitor (3-Deazaneplanocin) treatment assay (ranging from 0uM to 15uM) respectively. The p value significance is indicated as stars compared to control samples (*P < 0.05, **P < 0.005 and N/S, not significant). **(E)** ChIP assay showing the fold enrichment of EZH2, H3K27me3 and H2K27ac occupancy at *TET1* promoter using DAC treated and untreated MEC1 cell line.

When we looked for transcription factor occupancy at the *TET1* promoter in the UCSC genome browser in different cell lines from the ENCODE/Broad institute datasets, we found that EZH2 as one of the transcription factors significantly enriched at the *TET1* promoter (Figure 3A). We have also experimentally validated the presence of EZH2 binding to *TET1* promoter using ChIP-qPCR in four cell lines (MEC1, HG3, GRANTA 519 and Z138) (Supplementary Figure 2B), indicating a potential role of EZH2 in the transcriptional control of *TET1*. Considering the above observations that there is a regulatory connection at the post-transcriptional level between *miR26A1* and TET1/EZH2 and that EZH2 is enriched at the *TET1* promoter, we hypothesized that EZH2 may have functional role in *TET1* regulation at the transcriptional level. Towards this, we downregulated EZH2 in all the four cell lines using siRNA, and strikingly, downregulation of EZH2 led to significant decrease in *TET1* expression compared to control siRNA samples in all the four cell lines (Figure 3B and 3C). In addition, when MCL cell line samples were treated with increasing concentrations of EZH2 inhibitor DZNep there was a significant decrease in *TET1* levels along with *EZH2* (Figure 3D). We next wanted to investigate whether EZH2 has a direct role in the control of *TET1* expression. To investigate this we performed ChIP assay using EZH2 and H3K27me3 antibodies on MEC1 CLL cell line following DAC treatment. A significant reduction in EZH2 binding was observed at the *TET1* promoter in the DAC treated samples without change in the enrichment of EZH2 catalyzed H3K27me3, a repressive histone modification. Also, on TET1 promoter there was significant decrease in the enrichment of active histone acetylation mark, H3K27ac in DAC treated sample (Figure 3E). Taken together, these observations indicate that EZH2 directly controls the transcriptional activity of *TET1*.

### Identification of a novel cryptic promoter in the hypermethylated intronic region of TET1

**Figure 4:**
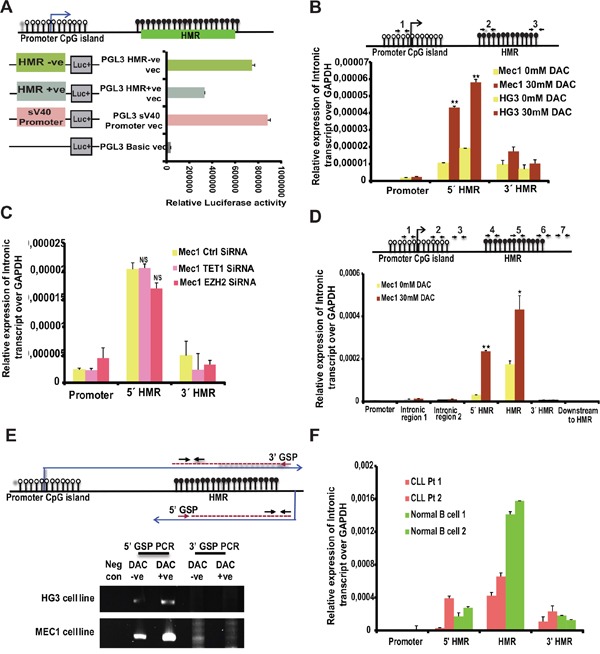
Promoter activity of HMR and presence of intronic transcripts within the *TET1* gene **(A)** The promoter activity of different PGL3 cloned vectors (as shown in the schematic diagram on left side of the graph) transfected in MCF-7 cell line analyzed using Dual luciferase reporter assay. HMR +ve and -ve indicates positive and negative orientation of the sequence cloned with respect to Luciferase gene. **(B)** Upper panel depicts the model illustrating the *TET1* promoter unmethylated CpG island (represented by white-filled lollipops) and HMR (represented by black filled lollipops) showing the location of three different primer sets (1, 2, and 3) used for below intronic transcript RT-qPCR. Below are the graphs showing the relative expression levels of intronic transcripts over *GAPDH* on the specified locations of TET1 gene for DAC treated untreated CLL cell line samples. **(C)** Relative expression levels of intronic transcript over *GAPDH* as mentioned above using EZH2 and TET1 siRNA samples compared to control siRNA transfected CLL cell line samples. **(D)** Graph showing the relative expression levels of intronic transcripts in MEC1 DAC treated and untreated samples using 7 different primer sets located between *TET1* peak region and promoter region of *TET1* gene. The location of these primer sets on *TET1* gene is indicated in the above illustrated diagram with numbers (1 to 7). **(E)** Detection of anti-sense intronic transcript of *TET1* gene using Reverse Transcription PCR. 5’ Gene specific primer (GSP) and 3’GSP are designed for specifically synthesizing cDNA from lower anti-sense strand and upper sense strand respectively. Lanes 1 to 5 shows the PCR amplified products from 5’GSP and 3’GSP cDNA synthesis using DAC treated and untreated CLL cell line samples (upper panel shows HG3 samples and lower panel shows MEC1 samples). The negative control indicates the sample with cDNA synthesized without any GSP primer. The location of GSP primers and the primer sets used for amplification after cDNA synthesis are indicated in the above illustrated schematic diagram. **(F)** Relative expression levels of intronic transcript over *GAPDH* using two CLL samples and two normal B cell samples at TET1 gene promoter and HMR regions.

From the published evidence it is known that promoter methylation correlates with gene repression [26] while the methylation of gene-body is linked to gene activation [27, 28]. More importantly, recent data demonstrate that gene-body methylation plays very important role in the transcriptional repression of cryptic promoters located in the intronic regions [22]. We wanted to check whether the hypermethylated region (HMR), which is around 685 bp (+1.75 to +2.4 kb from TSS), harbors any cryptic promoter activity in the absence of methylation. In order to check this possibility, the HMR region was amplified (632 bp) and cloned in both orientations into basic promoter-less luciferase vector. All the cloned vectors along with control basic vector (without any promoter) and a positive control vector (SV40 promoter) were transfected into CLL cell lines and analyzed for promoter activity. Strikingly, the HMR in negative orientation showed significantly high promoter activity, which is comparable to strong SV40 promoter activity (Figure 4A). However, the HMR fragment cloned in positive direction also showed promoter activity but significantly less than the HMR in negative orientation vector (Figure 4A).

Based on the promoter activity data of the HMR, we proceeded further to check for the presence of any intronic transcripts in the HMR region. To detect intronic transcripts encoded by the HMR region, the total cDNA was synthesized using DNase 1 treated total RNA and RT-qPCR analysis was performed using primers spanning, downstream and upstream of the HMR region. To check if amplification is from genomic DNA contamination, mock cDNA samples without RT enzyme were used as controls for all the respective cDNA samples. As shown in Figure 4B, RT-qPCR amplification detected transcripts from the HMR region and the expression levels of these transcripts were significantly induced in DAC treated samples compared to untreated samples in both CLL cell lines (Figure 4B). Later, when the same RT-qPCR analysis was done using EZH2 siRNA and TET1 siRNA CLL samples, there was no difference in the expression level of these transcripts (Figure 4C). To further investigate deeply different regions for the presence of transcripts across the HMR region, we designed total 7 sets of primers covering the region between the promoter TSS and the HMR and also downstream of the HMR. We could detect transcripts between the HMR region and upstream promoter TSS, but intensity of the transcripts decreased around the promoter TSS (Figure 4D). However, no transcription could be detected downstream of the HMR region, indicating that cryptic promoter from the HMR could be encoding transcripts in the antisense direction. To address the latter issue in more detail, we performed strand-specific cDNA synthesis using sense and antisense strand-specific primers that map region between the HMR and *TET1* promoter. As shown in Figure 4E, amplification was seen in the antisense strand-specific primer but not with the sense strand-specific primer. Amplification in the antisense strand-specific RT-qPCR was more in DAC treated MEC1 and HG3 samples compared to untreated samples, indicating that the loss of DNA methylation of the HMR induces cryptic transcripts (Figure 4E). We also performed strand-specific cDNA synthesis using control siRNA and EZH2 siRNA samples. Barring the DAC treated sample, which was used as a positive control, we could not detect any amplification of cryptic transcripts in control siRNA and EZH2 siRNA samples with sense and antisense strand-specific primers (Supplementary Figure 1E). Based on this data, one can expect that normal B cells would show increased expression levels of these intronic transcripts as HMR region is unmethylated. As shown in the Figure 4F, we see increased expression levels of intronic transcripts in normal B cells at HMR compared to CLL samples. Hence, this data shows that gene-body hypermethylation regulates the expression of cryptic promoters in HMR.

### Fine mapping the location of cryptic promoter and confirming the promoter activity

**Figure 5:**
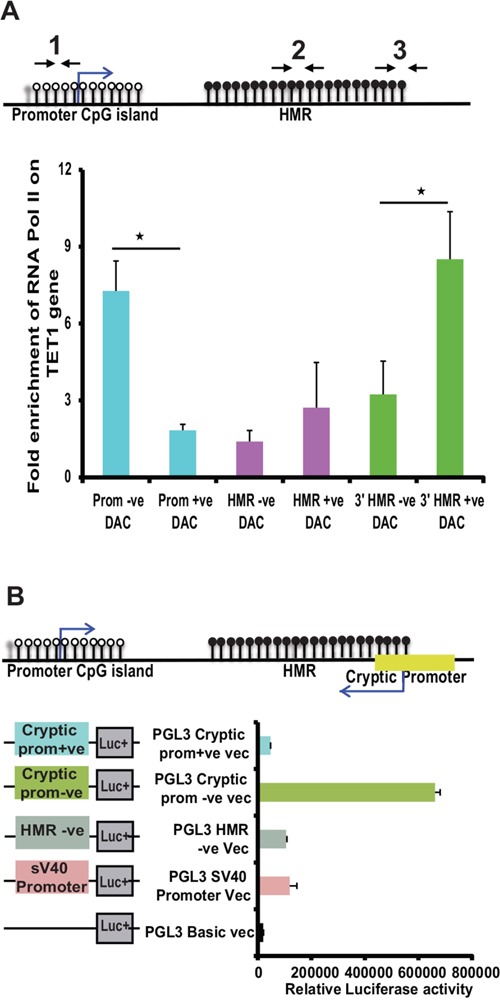
Analyzing the RNA Pol II occupancy and promoter activity at cryptic promoter region of TET1 gene **(A)** Fold enrichment of RNA pol II occupancy at TET1 promoter and cryptic promoter region (mapped to downstream of HMR) along with HMR on TET1 gene. The location of the ChIP primer sets (1 to 3) used for this assay is illustrated in the above diagram. **(B)** The promoter activity of different PGL3 cloned vectors (as shown in the schematic diagram on left side of the graph) transfected in MCF-7 cell line analyzed using dual luciferase reporter assay. The 519 bp predicted cryptic promoter region cloned for luciferase activity (green color bar) is shown in the schematic illustration above the graph.

Thus, our data clearly demonstrate the presence of a methylation dependent cryptic promoter at HMR located in the intron 1 of *TET1*, which encode antisense transcripts spanning across the HMR of its sense counterpart *TET1*. We next wanted to investigate whether induction of cryptic promoter activity has any functional connection to the *TET1* promoter activity upon DAC treatment. To this end, ChIP assay was performed on TET1 promoter TSS site, HMR region and downstream to HMR region using antibodies against RNA Pol II on DAC treated and untreated MEC1 cell line samples. Interestingly, following DAC treatment, the occupancy of RNA Pol II at the TET1 promoter decreases while at the HMR downstream region the RNA Pol II occupancy increases (Figure 5A). However, in the HMR region (middle region), there was no significant binding of RNA Pol II observed compared to the TET1 promoter and cryptic promoter regions. The exact locations of the ChIP primer sets used are shown as schematic diagram above the graph (Figure 5A).

Based on the RNA Pol II occupancy, ~500bp region (containing 3’ HMR and downstream to HMR sequence) was amplified containing the predicted cryptic promoter region and assayed for the promoter activity using luciferase assays. As shown in Figure 5B, the fragment containing predicted cryptic promoter showed higher luciferase activity in negative orientation, which is around 3 fold more compared to the earlier identified promoter activity of the HMR region.

## DISCUSSION

Genes are regulated at the levels of transcriptional initiation, transcription elongation, pre-mRNA 3’ processing and mRNA degradation. These gene regulatory steps further controlled by complex and coordinated multi-level cross talks between transcription factors, chromatin remodelers and long and small noncoding RNAs [29, 30]. In this investigation, we explored the functional role of miRNA, chromatin modifier and non-coding cryptic transcription in TET1 gene expression.

Gene-body hypermethylation has lately been recognized as a transcriptional regulatory step in regulating alternative splicing [31] and cryptic promoter silencing [32]. By definition, the promoter sequences that are located outside the defined promoter annotated regions in the genome are termed as cryptic promoters. They are in normal context often silenced in order to allow transcription initiation from the fully functional annotated promoters. Previously, by applying MBD-seq on normal B cells and CLL patients’ samples, we have identified differentially methylated region in the *TET1* gene-body [19]. Since *TET1* gene-body hypermethylation correlates with *TET1* gene activation, we wanted to understand the functional role of gene-body hypermethylation in *TET1* gene expression. DAC treatment to induce demethylation across the genome has been widely used to explore the functional role of DNA methylation in gene expression. Thus by treating four different CLL cell lines with DAC, we have shown that demethylation of hypermethylated *TET1* gene-body leads to the activation of methylation dependent cryptic promoter. Both strand-specific RT-PCR and promoter luciferase assays clearly demonstrated that cryptic promoter encodes antisense transcripts, which span across the *TET1* promoter. Activation of cryptic transcription across the *TET1* promoter correlates with the loss of RNA Pol II occupancy and *TET1* gene silencing, indicating that antisense cryptic transcription may be regulating *TET1* gene expression in part by occluding the transcription initiation machinery from the *TET1* promoter. Thus our data is consistent with the functional role of gene-body methylation in repressing aberrant cryptic promoter activity. Interestingly, we observed presence of many SINE and LINE Alu repeats located in the HMR region where the intronic transcripts are expressed. It would be interesting to know the role of these transcribed repetitive elements in TET1 expression.

Importantly, the DAC treatment assays also uncovered another interesting regulatory loop, involving *miR26A1*, EZH2, in the *TET1* gene expression. Previously, *miR26A1* has been implicated in the regulation of TET1 [25] and EZH2 [33]. Upon closer analysis of transcription factor occupancy at the *TET1* promoter in the UCSC genome browser utilizing the ENCODE/Broad Institute ChIP-seq datasets, revealed presence of EZH2 peaks at the *TET1* promoter in several cell types. Thus we wanted to investigate the functional interplay between EZH2, TET1 and *miR26A1*. Previously, we documented that *miR26A1* is hypermethylated in CLL samples compared to normal B cells [24]. Here we show that *miR26A1* can regulate *TET1* at the transcriptional level via modulating the levels of EZH2 at the *TET1* promoter. Interestingly, in this context EZH2 acts as an activator in the *TET1* gene regulation, which is in contrast to its widely accepted function as transcriptional repressor via catalyzing H3K27me3 modification. Previously, in a few instances EZH2 has been implicated in the gene activation function which is independent of histone methyltransferase activity [34, 35]. It remains to be seen whether *miR26A1* directly regulates *TET1* at the post-transcriptional level or via regulating EZH2 occupancy at the *TET1* promoter there by interfering with *TET1* activity indirectly at the transcriptional level. According to our data (Supplementary Figure 1E), depletion of EZH2 does not influence DNA methylation levels at the HMR and also TET1 transcripts levels, which rules out the functional role of EZH2 in TET1 transcriptional regulation via modulating the methylation of HMR.

**Figure 6:**
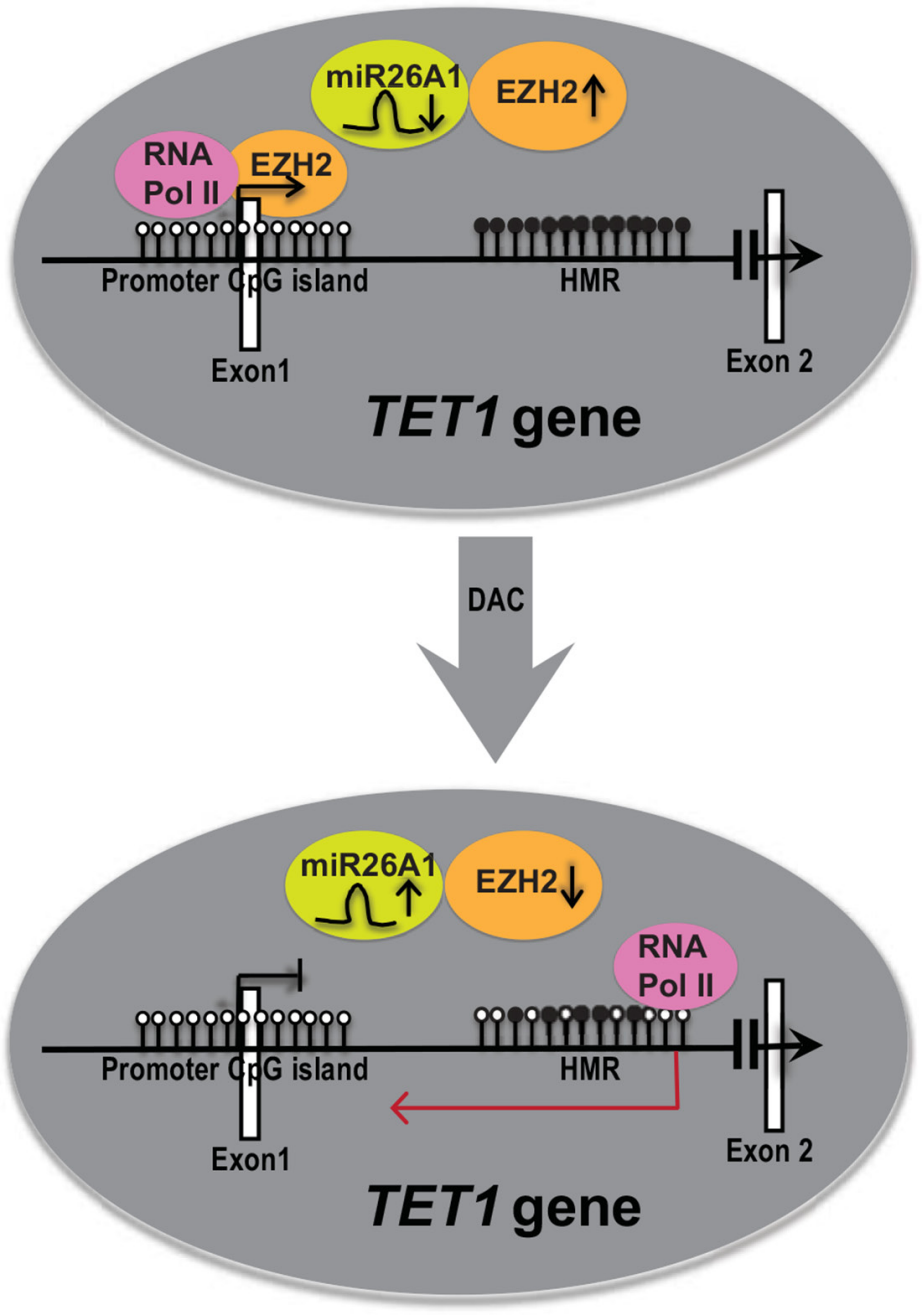
A model explaining the role of DNA hypermethylation in regulating TET1 gene expression The upper panel represents the expression status of *TET1* gene in CLL samples *in vivo*. The white and black filled lollipops represents unmethylated and methylated CpG sites on TET1 promoter CpG island and HMR CpG island in intronic region respectively. Each lollipop indicates one CpG site. The while colored rectangular boxes indicates exons. *miR26A1*, EZH2 and RNA Pol II are shown in colored circles with arrows up and down indicating higher and lower levels respectively. The above panel shows the representation of *TET1* expression before DAC treatment and the below arrow shows the representation of *TET1* after DAC treatment. In the upper panel, *TET1* gene is active and shown with open arrow. In the lower panel *TET1* gene is inactive and shown with closed arrow. The red color arrow in the anti-sense direction in the lower panel represents cryptic transcript. In the HMR of lower panel, few CpG sites methylated and few unmethylated depicting that this region is less methylated compared to above completely hypermethylated region.

Finally, a schematic representation of overall data of this study is shown in Figure 6. *TET1* is active and unmethylated at promoter region, but hypermethylated in the gene-body at intronic region (Figure 6, upper circle). Hypermethylation of HMR is required for the activation of the *TET1* gene as we observe decrease in *TET1* expression upon DAC (methyl inhibitor) treatment as shown in the Figure 6, lower circle. Loss of DNA methylation levels with DAC treatment resulted in the upregulation of *miR26A1*, decrease in EZH2 levels and induced expression of intronic transcripts at HMR region resulting in reduced RNA Pol II occupancy at the *TET1* promoter, leading to reduced *TET1* expression levels. Thus our data demonstrate a novel regulatory loop between *miR26A1*-EZH2 and TET1 and thus providing an explanation for consistent upregulation of TET1 and EZH2 in CLL patients while *miR26A1* is hypermethylated. Hence, our study opens up a new avenue in the further exploration of regulatory connection of EZH2 and TET1.

## MATERIALS AND METHODS

### CLL patient samples, cell culture conditions and transfections

Total 40 CLL patient samples were included in the present study. All the peripheral blood mononuclear cells (PBMCs) CLL samples were collected from different hematology departments in the western part of Sweden after written consent had been obtained. 5 DNA samples of CD+19 sorted normal B cells from healthy age matched controls were purchased from 3H biomedical (Uppsala, Sweden). All samples were diagnosed according to recently revised iWCLL criteria [36], showing typical CLL immunophenotype and 70% or more tumor percentage. The median age at diagnosis was 66 years (range, 41–85 years), with a male: female ratio of 3: 2. 20 CLL cases displayed IGHV mutated genes and 20 cases displayed IGHV unmutated genes. Four leukemic cell lines were used in this study, two CLL cell lines (HG3 and MEC1) [37, 38] and two MCL cell lines (Z138 and GRANTA 519) [39, 40]. The cell culture conditions and transfection methods performed in this study are described in Supplementary Materials.

### DNA, RNA extractions and cDNA synthesis

DNA was extracted from CLL PBMC samples using DNA extraction kit (Qiagen, Hilden, Germany) according to the manufacturer's protocol. RNA extractions were done using miRNeasy Mini Kit (Qiagen, Hilden, Germany) along with on column DNase1treatment (RNase free DNase 1 set, Qiagen, Hilden, Germany) to remove any residual genomic DNA. The total cDNA synthesis was performed using Superscript III FS synthesis supermix kit (Life technologies, Carlsbad, USA) according to the manufacturer's protocol.

### Pyrosequencing

The hypermethylated region (HMR) peak from obtained from MBD seq data [19] is 687 bp. Two sets of pyrosequencing primers were designed one in HMR region (252 bp, containing 8 CpG sites) and the other in the *TET1* promoter TSS (Transcriptional start site) (156 bp, containing 12 CpG sites). All the primer sequences used for Pyrosequencing assay are listed in Supplementary Table 1. The pyrosequencing was performed as described earlier [41] and is described briefly in Supplementary Materials.

### Quantitative RT-PCR and strand specific RT-PCR

The expression levels of TET1 and EZH2 genes were analyzed with Taqman gene expression assays (Applied Biosystems, Warrington, UK) (Hs00286756_m1 for TET1, Hs01016789_m1 for EZH2 and Hs99999905_m1 for Glyceraldehyde-3-phosphate dehydrogenase, which was used as an internal control). For the expression of intronic transcripts, 7 sets of custom primers were designed using Primer 3 software, spanning the intronic region between Promoter TSS and HMR on TET1 gene and the RT-qPCR analysis was done using Power SYBR Green PCR master mix (Applied Biosystems, Warrington, UK). Differences in expression were calculated using the ΔΔCt method. All the primer sequences and the product size of the amplified region were listed in the Supplementary Table 1.

Strand specific PCR is performed using specifically designed 5’GSP (Gene specific Primer) and 3’GSP for synthesizing gene specific cDNA from DNase1 treated RNA samples. The 5’GSP specifically binds to antisense strand and the 3’GSP binds to the sense strand. After cDNA synthesis, to quantify the strand specific cDNA synthesis we used two sets of primers which are located downstream to the 5’GSP and 3’GSP. The PCR product size and the primer sequences used were listed in Supplementary Table 1. The PCR conditions for amplification were 95°C for 10mins, 95°C for 30seconds, 55°C for 30seconds, 72°C for 30seconds and 30 cycles of 95°C, 55°C and 72°C for 30seconds and finally 72°C for 3minutes. The amplified product was run in 1% agarose gel and visualized using the ChemiDoc XRS+ (Bio-Rad) instrument.

### Cloning and luciferase reporter assays

In order to identify the promoter activity of HMR and downstream cryptic promoter regions, we constructed several PGL3 dual-luciferase reporter vectors (Promega, Madison, WI, USA) containing the target sequence for checking the promoter activity. Luciferase activity was determined 48 h after transfection as described earlier [42], by use of the dual-luciferase reporter assay system (Promega, Madison, USA) in duplicate samples, according to the protocol supplied by the manufacturer. Detailed protocol is included in the Supplementary Materials.

### ChIP assay, western blot analysis, DNA methyl inhibitor and EZH2 inhibitor drug treatment

ChIP was performed with the shearing module kit (Diagenode, Liege, Belgium) and one-day ChIP kit (Diagenode, Liege, Belgium), according to the manufacturer's instructions. Western blot analysis was performed using total cell lysates lysed from transfected CLL cell line samples. The details of ChIP assay, western blot analysis along with the Antibodies used are described in Supplementary Materials and Primer sequences are provided in Supplementary Table 1. To investigate the effect of CpG methylation on gene expression, cells were treated with the 5’-Aza-2’-deoxycytidine (DAC) methyl inhibitor (Sigma-Aldrich, St. Louis, USA) using CLL cell lines cultured in RPM1 media as described previously [17]. The down-regulation of EZH2 in MCL cell lines was done as described earlier [43], using EZH2 inhibitor, 3-Deazaneplanocin A (DZNep) (Cayman chemicals, Michigan, USA) for three days using different concentrations ranging from 0–15 μM.

## SUPPLEMENTARY MATERIALS FIGURES AND TABLES


