# Supplementary material for: Gene-body hypermethylation controlled cryptic promoter and miR26A1-dependent EZH2 regulation of TET1 gene activity in chronic lymphocytic leukemia
Source: Oncotarget. 2017 Sep 6;8(44):77595–608. doi: 10.18632/oncotarget.20668 (PMC5652802; doi:10.18632/oncotarget.20668)
Supplement: Supplementary file 1 [file oncotarget-08-77595-s001.pdf]

Gene-body hypermethylation controlled cryptic promoter and miR26A1-dependent EZH2 regulation of TET1 gene activity in chronic lymphocytic leukemia

SUPPLEMENTARY MATERIALS

Cell culture conditions and transfection assays

The cell lines were cultured in RPMI 1640 media supplemented with 10% fetal bovine serum (FBS; Sigma-Aldrich), 4mM glutamine, and 1x Penicillin/streptomycin (Sigma-Aldrich, St. Louis, USA). MCF-7 cells were cultured in the presence of Dulbecco's modified eagle media (Gibco, Waltham, USA) with 10% heat inactivated FBS (Sigma-Aldrich, St. Louis, USA) and 1x penicillin-streptomycin (Sigma-Aldrich, St. Louis, USA).

The siRNA transfections and over expression assays, were performed using custom designed EZH2 siRNA and negative control siRNA (Invitrogen, Carlsbad, USA), miR26A1 mimic miRNA and miR26A1 inhibitor miRNA (Thermoscientific, Waltham, USA). Transient transfection was carried out on an Amaxa Nucleofection Device II (Nucleofector 2b device, Lonza group AG, Basel, Switzerland) according to the manufacturer instructions. The Luciferase assays were performed in MCF-7 cells, using Lipofectamine LTX plus reagent kit (Invitrogen, Carlsbad, USA) according to manufacture instructions.

Pyrosequencing assay

Pyrosequencing was performed according to the manufacturer's instructions using the PyroMark™ Q24 Advanced pyrosequencer (Qiagen, Hilden, Germany). The Genomic DNA was bisulfite converted using the EZ DNA Methylation Gold kit (Zymo Research, Irvine, USA) according to manufacturer's protocol. The PyroMark™ software (Qiagen, Hilden, Germany) was applied to design pyrosequencing primer sets. The percentage of DNA methylation (average value for all the CpG sites in the target region) used for statistical analysis was calculated using PyroMark Q24 advanced software. The statistical analysis and the calculation of p value for comparison between CLL samples and normal controls were done using Statistica12.0 (Stat Soft, Tulsa, USA).

Luciferase reporter assays

First, we amplified HMR region (632bp) and fine mapped cryptic promoter TSS region containing region (519bp) using primer sequences anchoring restriction sites for cloning these fragments directly into PGL3 multiple cloning sites in both orientations. The 5'-ends of the forward primers were added using the *Kpn1* restriction site and the 5'-ends of reverse primers were added using the *Xho*I restriction site and for reverse orientation it is vice versa (primer sequences shown in Supplementary Table 1). Two control vectors were used for transfections along with the cloned fragment vectors; positive control vector containing strong SV40 promoter (Promega, Madison, WI, USA) and the negative control vector without any promoter (Promega, Madison, WI, USA). The entire cloned sequence of the amplified fragments of PGL3 Luciferase reporter vectors were verified by sanger sequencing before transiently transfected using Lipofectamine LTX plus reagent kit (Invitrogen, Carlsbad, USA) in MCF-7 cells according to manufacture instructions in the presence of the β-galactosidase reporter gene (Promega, Madison, USA).

The relative light units were measured with a luminometer (Glomax 20/20 luminometer, Promega, Madison, USA). Each experiment was repeated for at least three independent sets, and the final luciferase values (relative light units) were calculated by dividing the luciferase activity by the β-galactosidase activity. Data represent average ± standard deviation (SD) of three independent experiments performed in triplicates.

ChIP assay

In brief, the transfected cells were formaldehyde-crosslinked, lysed, and sonicated four times for 10 min each (30s on / 30 s off) with bioruptor and the Shearing module kit. The final eluted DNA was analyzed by real-time quantitative PCR with the Power SYBR Green PCR master mix (Applied Biosystems, Warrington, UK) for the presence of the EZH2, H3K27me3, RNA pol II binding sites on TET1 promoter and HMR regions. Antibodies used for the ChIP assay were: EZH2 polyclonal antibody (pAb-039-050, Diagenode, Liege, Belgium), H3K27Ac polyclonal antibody (C15410174, Diagenode, Liege, Belgium), H3K27me3 polyclonal antibody (C15410089, Diagenode, Liege, Belgium) and RNA Pol II monoclonal antibody (C15200004, Diagenode, Liege, Belgium).

The PCR analysis was performed with the 7900HT fast real-time PCR system instrument and software (Applied Biosystems, Warrington, UK). The average percentage of input and fold enrichment was calculated which represents the enrichment of histone modification or binding of transcription factor on specific region of TET1 gene using the ChIP reactions performed in triplicates.

Western blot analysis

Western blot analysis was performed using total cell lysates lysed from transfected CLL cell line samples using RIPA buffer (Sigma-aldrich, St.Louis, USA) with PI inhibitors (Roche, Basel, Switzerland). Equal amounts of lysates were loaded on NUPAGE 10% Bis-Tris gels (Invitrogen, Carlsbad, USA) and transferred to membranes (Amersham Hybond ECL; GE Health Care Life Sciences, Sweden). After blocking in 5% BSA with TBS with addition of 0.1% Triton X-100, the membranes were incubated with the appropriate primary and secondary antibodies, followed by washes with TBS containing 0.05% Triton X-100. Blots were visualized with SuperSignal West Dura Extended Duration Substrate (Thermo Scientific, Rockford, USA) using the ChemiDoc XRSC (Bio-Rad) instrument. The primary antibodies used for western blotting were: TET1 (ab191698, Abcam, Cambridge, UK), EZH2 (3147, Cell Signaling Technology, Danvers, USA) and GAPDH (SC-25778; Santa Cruz Biotechnology, Dallas, USA), while the secondary antibodies were anti-mouse IgG (7076S, Cell Signaling Technology, Danvers, USA) and anti-rabbit IgG (7074S, Cell Signaling Technology, Danvers, USA).

TET1 expression using RNA sequencing data obtained from published CLL data set

We obtained the raw data of RNA-seq samples for 96 patients along with 9 normal B cell samples as described in our earlier paper [1]. In short, the obtained gene expression profile was normalized to reads per kilobase of transcript per million mapped reads (RPKM). The log-fold changes between B cell normal and two CLL groups (IGHV-mutated and IGHV-unmutated) were calculated based on obtained RPKM values. Wilcoxon rank sum test in R package is used for calculating statistical p value for differential expression between normal and CLL prognostic groups.

REFERENCE

1. Subhash S, Andersson PO, Kosalai ST, Kanduri C, Kanduri M. Global DNA methylation profiling reveals new insights into epigenetically deregulated protein coding and long noncoding RNAs in CLL. Clin Epigenetics. 2016; 8:106.

Supplementary Table 1: List and details of all the primer sequences used in this study

Gene	Primer type	Primer sequences (5'-3')
TET1 promoter	Pyrosequencing primers (156 bp)	5'GGGGGTTAGGGTTTTGATTGT 3' (Forward primer)
		(Biotin) 5'CCCCCAACTCCAAACCTAC 3'(Reverse primer)
		5' GGGTTTTGATTGTGTTG 3' (Sequencing primer)
TET1 HMR	Pyrosequencing primers (252 bp)	5'AGATTTTGGAGAGAGAATAGGATTAGGTA3' (Forward primer)
		(Biotin) 5'ATTCCTAATATACTCCAAATAACTATTACC 3'(Reverse primer)
		5' GAGAATAGGATTAGGTATATAG 3' (Sequencing primer)
TET1 promoter	ChIP primers (157 bp)	5' GACCTTTGGGAACCGACTC3'(Forward primer)
		5' CTCGGGCAAACTTTCCAACT 3' (Reverse primer)
HMR	ChIP primers (207 bp)	5' AAGACCTTGGAGCGAGAACA 3' (Forward primer)
		5' CGCCTTTTCCATATTTTTCG 3' (Reverse primer)
TET1 HMR amplification (+ve orientation)	PCR primers for amplification with KPN1 and Xho1 anchored sites (632 bp)	5' AAAGGTACC TAAAGCTTCAGCCTCGGAGT 3' (Forward primer)
		5' AAACTCGAGTCCCTTAATTGGGGAAAAGG 3' (Reverse primer)
TET1 HMR amplification (-ve orientation)	PCR primers for amplification with KPN1 and Xho1 anchored sites (632 bp)	5' AAACTCGAGTAAAGCTTCAGCCTCGGAGT 3' (Forward primer)
		5' AAAGGTACC TCCCTTAATTGGGGAAAAGG 3' (Reverse primer)
5' HMR	Intronic transcript PCR primers (129 bp)	5' TAAAGCTTCAGCCTCGGAGT 3' (Forward primer)
		5' GTTTAGTGCCCAGGAGTGGA 3' (Reverse primer)
3' HMR	Intronic transcript PCR primers (198 bp)	5' GGTGGCTTTTGGTGTGTTTC 3' (Forward primer)
		5' GCCTGGACTCCTCATCTTTG 3' (Reverse primer)
HMR	Intronic transcript PCR primers (207 bp)	5' AAGACCTTGGAGCGAGAACA 3' (Forward primer)
		5' CGCCTTTTCCATATTTTTCG 3' (Reverse primer)
Intronic region 1	Intronic transcript PCR primers (197 bp)	5' GGGGTGAGGAGAGGCTAGAG 3' (Forward primer)
		5' CCACTCCAAACGTTTCATCC 3' (Reverse primer)
Intronic region 2	Intronic transcript PCR primers (137 bp)	5' GGAGTTGGCTGGGAGTTGTA 3' (Forward primer)
		5' ACTGATGGGATCGCAAGG 3' (Reverse primer)
Downstream to HMR	Intronic transcript PCR primers (205 bp)	5' ACAACTCTGGGACTGCCACT 3' (Forward primer)
		5' AAGGGGATAGGGGAAATCAA 3' (Reverse primer)
5' GSP	Strand specific PCR primer	5' TAAAGCTTCAGCCTCGGAGT 3'
3' GSP	Strand specific PCR primer	5' GCCTGGACTCCTCATCTTTG 3'
Antisense strand PCR	Antisense strand amplification primers (198 bp)	5' GGTGGCTTTTGGTGTGTTTC 3' (Forward primer)
		5' GCCTGGACTCCTCATCTTTG 3' (Reverse primer)
Sense strand PCR	Sense strand amplification primers (129 bp)	5' TAAAGCTTCAGCCTCGGAGT 3' (Forward primer)
		5' GTTTAGTGCCCAGGAGTGGA 3' (Reverse primer)
TET1 cryptic promoter amplification (+ve orientation)	PCR primers for amplification with KPN1 and Xho1 anchored sites (519 bp)	5' AAAGGTACCGTGGCTTTTGGTGTGTTTCC 3' (Forward primer)
		5' AAACTCGAGAAAACTGAGGAAGGGCAAAAA 3' (Reverse primer)
TET1 cryptic promoter amplification (-ve orientation)	PCR primers for amplification with KPN1 and Xho1 anchored sites (519 bp)	5' AAACTCGAGGTGGCTTTTGGTGTGTTTCC 3' (Forward primer)
		5' AAAGGTACCAAAACTGAGGAAGGGCAAAAA 3' (Reverse primer)

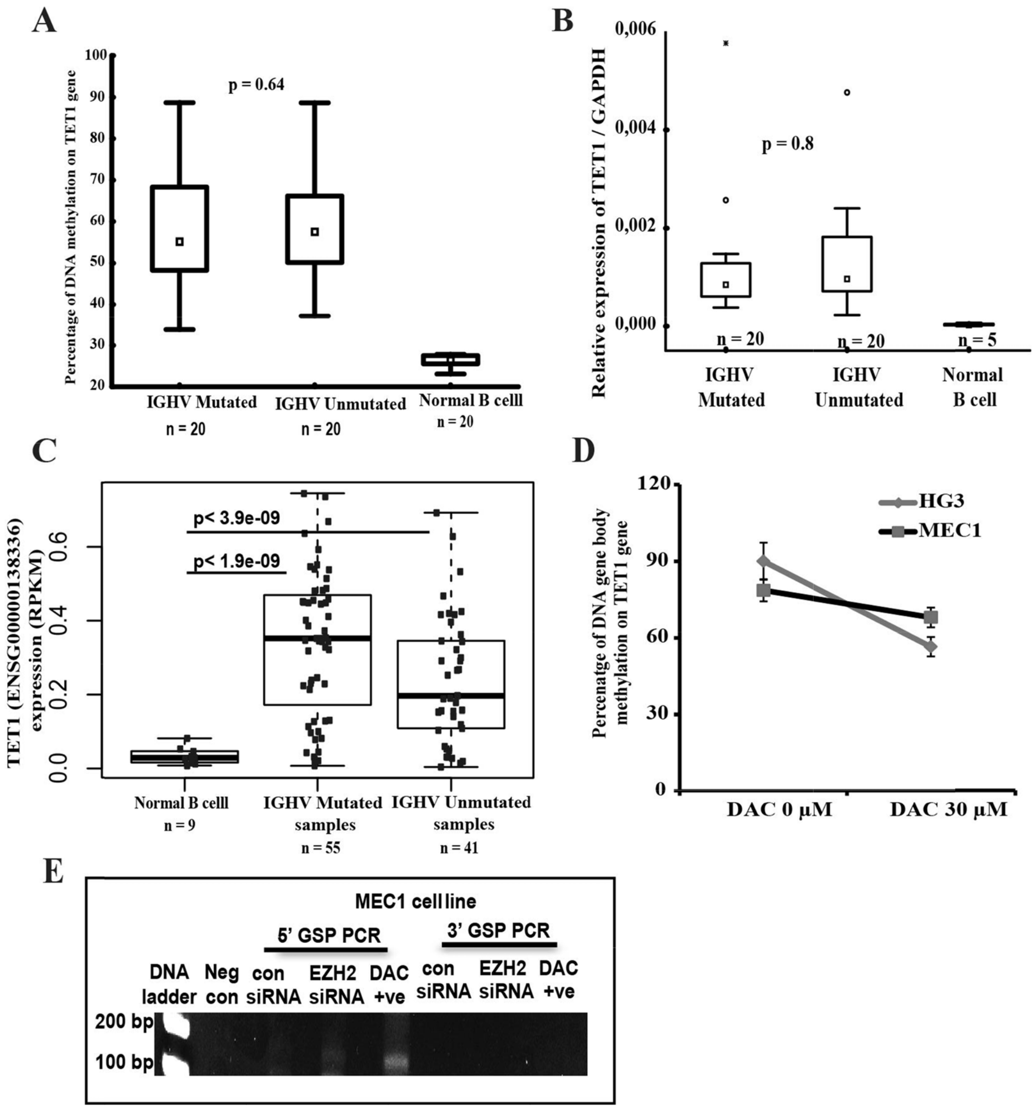

Supplementary Figure 1: DNA methylation status and expression levels of TET1 gene. (A) Box plots showing the percentage of DNA methylation on CpG sites at HMR of *TET1* gene using pyrosequencing in IGHV mutated, IGHV unmutated CLL samples compared with sorted normal B cells. **(B)** Box plots showing relative expression levels of *TET1* over *GAPDH* in CLL and normal B cell samples using RT-qPCR. **(C)** The boxplots shows the difference in gene expression levels between IGHV mutated, IGHV unmutated and sorted B cells for *TET1* gene obtained using published RNA sequencing dataset. **(D)** The percentage of DNA methylation at HMR of *TET1* gene using DAC treated and untreated MEC1 and HG3 CLL cell lines. **(E)** Detection of anti-sense intronic transcripts using strand specific Reverse Transcription PCR. 5'GSP and 3'GSP are designed for specifically synthesizing cDNA from lower anti-sense strand and upper sense strand respectively. Lanes 1 to 7 shows the PCR amplified products from 5'GSP and 3'GSP cDNA synthesis using control siRNA and EZH2 siRNA MEC1 cell line samples.

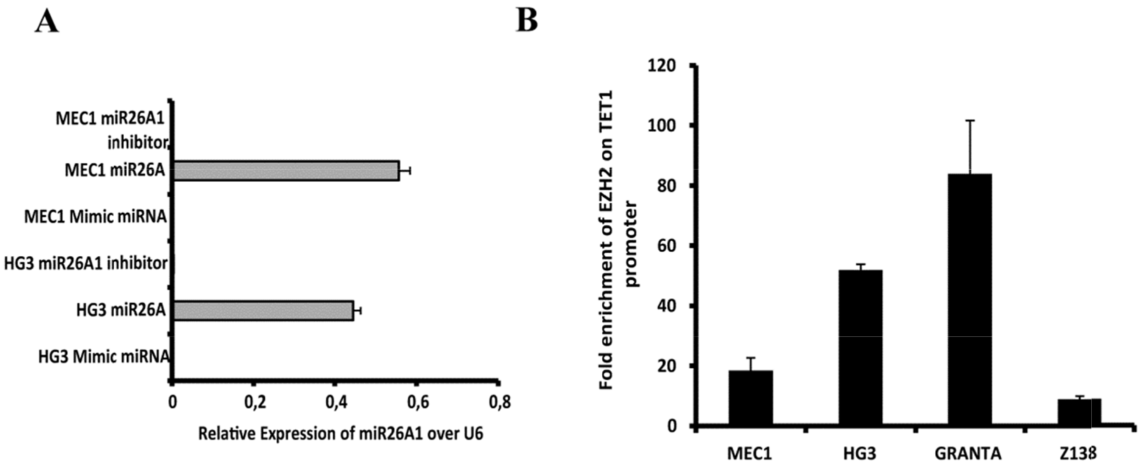

Supplementary Figure 2: Expression of miR26A1 and occupancy of EZH2 at TET1 promoter. **(A)** Relative expression levels of *miR26A1* in *miR26A1* and *miR26A1* inhibitor overexpressed HG3 and MEC1 cell lines compared to control mimic microRNA sample. *U6* is used as internal control. **(B)** Fold enrichment of EZH2 levels on TET1 promoter over IgG in all the four leukemic cell lines.
